# Influence of Physicochemical Characteristics and Stability of Gold and Silver Nanoparticles on Biological Effects and Translocation across an Intestinal Barrier—A Case Study from In Vitro to In Silico

**DOI:** 10.3390/nano11061358

**Published:** 2021-05-21

**Authors:** Yvonne Kohl, Michelle Hesler, Roland Drexel, Lukas Kovar, Stephan Dähnhardt-Pfeiffer, Dominik Selzer, Sylvia Wagner, Thorsten Lehr, Hagen von Briesen, Florian Meier

**Affiliations:** 1 Fraunhofer Institute for Biomedical Engineering IBMT, 66280 Sulzbach, Germany; michelle.hesler@ibmt.fraunhofer.de (M.H.); sylvia.wagner@ibmt.fraunhofer.de (S.W.); hagen.briesen@ibmt.fraunhofer.de (H.v.B.); 2 Postnova Analytics GmbH, 86899 Landsberg am Lech, Germany; roland.drexel@postnova.com; 3 Department of Clinical Pharmacy, Saarland University, 66123 Saarbrücken, Germany; lukas.kovar@uni-saarland.de (L.K.); dominik.selzer@uni-saarland.de (D.S.); thorsten.lehr@uni-saarland.de (T.L.); 4 Microscopy Services Dähnhardt GmbH, 24220 Flintbek, Germany; sd@msd.sh

**Keywords:** metallic nanoparticles, shape, zeta potential, nano-bio interactions, in vitro studies, translocation study, gastrointestinal barrier, in silico modeling, nanotoxicology, nanosafety

## Abstract

A better understanding of their interaction with cell-based tissue is a fundamental prerequisite towards the safe production and application of engineered nanomaterials. Quantitative experimental data on the correlation between physicochemical characteristics and the interaction and transport of engineered nanomaterials across biological barriers, in particular, is still scarce, thus hampering the development of effective predictive non-testing strategies. Against this background, the presented study investigated the translocation of gold and silver nanoparticles across the gastrointestinal barrier along with related biological effects using an in vitro 3D-triple co-culture cell model. Standardized in vitro assays and quantitative polymerase chain reaction showed no significant influence of the applied nanoparticles on both cell viability and generation of reactive oxygen species. Transmission electron microscopy indicated an intact cell barrier during the translocation study. Single particle ICP-MS revealed a time-dependent increase of translocated nanoparticles independent of their size, shape, surface charge, and stability in cell culture medium. This quantitative data provided the experimental basis for the successful mathematical description of the nanoparticle transport kinetics using a non-linear mixed effects modeling approach. The results of this study may serve as a basis for the development of predictive tools for improved risk assessment of engineered nanomaterials in the future.

## 1. Introduction

When manufacturing new products that contain engineered nanomaterials (ENM), often referred to as nano-enabled products [1], in addition to improving product functionality and quality, the safety of the products for the user as well as safe manufacturing (safety of workers) must be ensured. The topic of safe-by-design (SbD) is therefore gaining more and more importance in the field of nanotechnology [2,3,4,5]. SbD is not a new concept; it has already been applied by other industries, among others also in the pharmaceutical sector to ensure safety throughout the drug discovery and development process [4,6,7,8]. However, its implementation in nanotechnology remains challenging and requires a comprehensive and well-founded database for functionality, toxicity and exposure of ENM. When it comes to the development of safe ENM, a profound understanding of their functionality is likewise necessary [9]. In order to develop safe nano-enabled advanced materials, it is essential to know the potential exposure scenarios of the ENM in the foreseen final product, to collect critical physicochemical properties that influence the ENM functionality and to identify basic toxicological information at the earliest possible stage of product development. Key parameters that influence ENM exposure include e.g., stability, release, toxicity and the relationship between these parameters and associated human or environmental risks are still not well understood. Knowledge gaps between ENM application and safety assessments still exist and need to be closed by new strategies including screening technologies, as well as a combination of screening systems and prediction models as a basis for new SbD concepts [10]. However, such SbD concepts rely on the availability of sufficient and reliable experimental studies and data sets. In this context, this study focuses on the influence of the physicochemical characteristics and the stability of metallic nanoparticles (mNP), in particular gold nanoparticles (AuNP) and silver nanoparticles (AgNP) of different sizes, shapes and surface zeta potentials, on the translocation through the gastrointestinal (GI) barrier, along with their associated biological effects, such as membrane damage, inflammation, apoptosis or genotoxicity.

Due to their diverse physicochemical characteristics mNP are key components of innovations in various fields with high potential impact (e.g., energy generation and storage, electronics, photonics, diagnostics, theranostics, antimicrobial applications or drug delivery agents) [11,12]. To ensure the safety for humans and the environment, it is important to investigate key characteristics that influence the release, exposure, behavior in the environment, and effects of these mNPs on organisms in order to establish an improved risk assessment.

In general, the human body can come into contact with ENM through three main uptake routes: inhalation, ingestion and absorption [13]. In case of ingestion, the intestinal mucus, a complex network of highly branched glycoproteins, lipids, cellular and serum macromolecules, is the first barrier, which ingested ENM must pass [14]. The mucus can entrap ENM because it poses a physical barrier due to its thickness, density, negative charge and constant renewal [14,15,16] with ENM surface charge and size influencing mucus adhesion and penetration [17]. Typically, the most common mechanism for uptake of ENM into intestinal epithelial cells appears to be endocytosis after overcoming the mucosal barrier [17]. pH variance within the GI compartments can affect aggregation status and alter surface chemistry of the ENM, as ζ-potential is highly pH-dependent [18]. After ingestion, and also in an in vitro environment, ENM develop a protein corona, consisting of a mix of proteins, small molecules and ions that adsorb to the particle surface [19,20,21,22,23,24]. Bare mNP added into the blood system are quickly opsonized by plasma proteins [25]. Binding of polymers on the surface of the ENM protects them from opsonization, determines their final size, controls their growth, changes their surface charge and thus the interaction with the negatively charged cell membrane, prevents clustering or agglomeration and helps to reduce their toxicity [26,27]. Such surface modifications influence the bioavailability und functionality of ENM, which enables longer half-life in the blood stream, improved biodistribution and higher ENM stability [26]. For example, AuNP with albumin surface modification show lower toxicity, longer blood circulation time, as well as better bioavailability and selective bioaccumulation [26].

Besides the biological features of the GI tract, the physicochemical features of the ENM strongly impact data interpretation from ENM ingestion experiments. Particle size and size distribution, particle number and surface area, as well as aggregation/agglomeration state, surface charge, shape and stability are all likely to influence the biological availability and effects of an administered ENM [13,28,29,30]. In the literature, many different types of AuNPs are described, varying in size, shape (e.g., sphere, shell, star, rod, cube, diamond) or surface modification [11]. Oxidant generation and rate of dissolution will also impact absorption and biological response [31].

Mechanisms of how ENM enter the GI cells and interact with cellular structures are still not fully understood [32,33,34]. Besides studies on particle modifications and characteristics during digestion using digestion models (mimicking gastric or small intestinal conditions) [35,36,37,38,39], several in vitro studies had focused on ENM translocation and uptake behavior with different GI models, ranging from very simple monolayer cultures, up to models mimicking the GI environment as realistically as possible, to generate physiologically relevant results. Among the variety of cell models available in vitro, the human epithelial colorectal adenocarcinoma cells Caco-2 are the most commonly used in ENM translocation studies [40]. The reason for that is their expression of tight junctions. Several examples are described in the literature where monocultures of Caco-2 cells have been used to study the in vitro translocation of ENM (mainly polystyrene but also silicon, silver and organic nanoparticles) [41,42,43,44]. A potential drawback of Caco-2 monolayers is the lack of a mucus layer, which can, however, be introduced by co-culturing Caco-2 cells with the human mucus-secreting colon adenocarcinoma cells HT29-MTX [41,43,45,46]. A further cell type which is often combined with GI mono- or co-cultures are epithelial cells of the GI, which are part of the immune system. The so-called M cells, or Raji cells, are combined with Caco-2 cells as co-culture or as triple culture with both, Caco-2 and HT29-MTX [43,47,48,49,50,51]. Depending on the used in vitro model, different EC_50_ values have been determined for ENM, e.g., AgNP in the size range of 20 nm [47,52,53,54].

Until now, only a few studies have investigated the correlation between the ENM physicochemical properties and their influence on the GI translocation in a triple co-culture model.

Many of the published studies with AuNP or AgNP use monocultures or co-cultures of two cell types. These show that the composition of the cell models, the incubation conditions, including duration and the investigated dose have an influence on the induced effects [47,55,56,57]. Qin et al. (2020) used the Caco-2 model and analyzed the proteomic composition of the intracellular protein corona of AuNP, which enabled the tracing of transport pathways in the epithelial cells [58]. Kämpfer and co-workers (2020) used a co-culture model consisting of Caco-2 and THP-1 cells, mimicking the intestine in a healthy and inflamed state for nanotoxicological research [59] while Mortensen et al. (2020) [7] tested a range of metal, metal oxide and metal sulphide ENM to study their influence on the intestinal barrier function and their cytotoxicity in Caco-2 cells.

Moreover, in vivo animal studies have shown that AuNP are able to enter the organism after oral ingestion. The AuNP circulate in the blood stream and accumulate in various organs such as liver, spleen and lymph nodes [32,60]. A size-dependent biodistribution was measured by Hillyer et al. 2001 [61]. In contrast to AuNP, AgNP increased intestinal permeability and caused inflammation of intestinal tissue [62].

Braakhuis and colleagues recently suggested that modeling experimental data of nanoparticles with in silico techniques could help bridge the gap between ENM in vitro translocation and in vivo bioavailability in the future [44]. For this purpose, the physiological nature of in vitro models needs to be increased and new in silico models need to be developed [44]. Various compartmental modeling approaches have proven to be valuable tools in modeling cell permeation of small molecules [63]. As AuNP have shown to be able to translocate through cell layers [64] and taken up by the cell [65,66], compartmental modeling could also be a helpful technique in characterizing the translocation kinetics of different ENM species [67].

In the present study, a combined approach of detailed physicochemical analysis, toxicological assays and in silico modeling was used to answer the question on the influence of the physicochemical characteristics and stability of AuNP on their translocation across the intestinal barrier and related biological effects. Using AuNP of different sizes and shapes (spheres with a mean diameter of 30 nm and 200 nm, rods with a mean diameter of 40 nm and a mean length of 112 nm) corona formation in cell culture medium, surface chemistry and further physicochemical parameters are correlated with their translocation properties, while AgNP were used for comparative purposes.

## 2. Materials and Methods

### 2.1. Nanomaterials

Gold nanospheres with a mean diameter of 30 nm (product code EM.GC 30/4) and 200 nm (product code EM.GC 200/4) were purchased from BBI Solutions (Crumlin, UK). Gold nanorods (AuNRods) with a length of 112 nm and a diameter of 40 nm were purchased from Nanopartz™ (A12-40-750-CIT-DIH-1-25-CS-EP, Loveland, CO, USA). AgNP, spheres with a mean diameter <20 nm (NM-300K, JRC) were obtained from the Fraunhofer Institute for Molecular Biology and Applied Ecology IME (Schmallenberg, Germany). All ENM stock suspensions were treated in an ultrasonic bath (Elmasonic S15, Elma, Germany) for 10 min to disrupt agglomerations, before mixing with pre-warmed (37 °C) cell culture medium (CCM) to create the test concentrations. Testing concentrations were defined as 30 µg/mL (EC_50_) for the positive control AgNP and 1 µg/mL for the test substances AuNP (spheres and rods). A concentration of 1 µg/mL was chosen to cover two aspects; on the one hand, to approach the ppm range (environmental relevant) and to further assure that the cell barrier is not impaired which would affect the translocation analysis.

### 2.2. Determination of Size Distribution and Ionic Concentration via Single Particle Inductively Coupled Plasma Mass Spectroscopy (spICP-MS)

Ultrapure water (UPW, resistance 18.2 MOhm) was obtained from a MilliQ system (Integral 5 system, Merck, Darmstadt, Germany). For sample preparation and dilution, TritonX100 (J.T. Baker Mallinckrodt, Deventer, The Netherlands) and an aqueous solution of Tetramethylammonium Hydroxide (TMAH) (25% (*v/v*), AppliChem, Darmstadt, Germany) were purchased. All spICP-MS measurements were carried out with a diluent of 0.1% TritionX100 and 0.5% TMAH. Furthermore, hydrochloric acid (HCl) (30% (*w/w*)) and nitric acid (HNO_3_) (65% (*w/w*)) were both acquired in suprapur quality from Merck (Darmstadt, Germany).

Ionic ICP standards of silver and gold with a stock concentration of 1000 mg/L (Carl Roth, Karlsruhe, Germany) were purchased to construct a calibration line. The relationship between spICP-MS count rate and concentration was established by using different concentrations of ionic silver and gold standards diluted in the same diluent as the analytes. For determining the nebulization efficiency (transport efficiency), a reference suspension of AuNP with a nominal diameter of 58 nm was used (product code EM.GC60, BBI Solutions, Crumlin, UK). The nebulization efficiency was calculated according to the “size method” published by Pace et al. (2011) [68] using the nominal the size determined via transmission electron microscope.

spICP-MS measurements were carried out on an Agilent ICP-MS 7900 (Agilent Technologies, Waldbronn, Germany). The samples were introduced using an ASX-500 Autosampler (Agilent Technologies, Waldbronn, Germany) and a peristaltic pump operating at 0.1 rpm, which corresponds to a flow rate of 0.346 mL/min. The sample introduction system consisted of a MicroMist nebulizer (Agilent Technologies, Waldbronn, Germany), a quartz glass spray chamber (Scott double-pass, Agilent Technologies, Waldbronn, Germany) and a quartz glass torch (2.5 mm ID injector, Agilent Technologies). The spray chamber was cooled to 2 °C. A nebulizer gas flow rate of 1.09 L/min, a plasma gas flow rate of 15 L/min and an auxiliary gas flow rate of 0.9 L/min were applied for analysis. All inserted gas flows consisted of Argon gas (purity 5.0) and all experiments were conducted without using any collision gas. A radio frequency power of 1550 W and a sampling depth of 6.0 mm were used throughout all experiments. The dwell time was set to 100 µs without any settling time. Data were acquired for 60 s recording the intensities of the isotopes ^107^Ag and ^197^Au, respectively. A tune operation according to the manufacturer’s recommendations was performed on a daily basis to ensure optimal hardware functionality and alignment. Prior to analyte measurements the nebulization efficiency and element response calibration were calculated. The nebulization efficiencies ranged between 4.8% and 6.2%.

All standards and samples were diluted using a mixture of 0.5% TMAH and 0.1% TritonX100 to ensure comparable and stable conditions as well as nebulization efficiencies [69,70]. Dilution factors of up to 10^6^ were required to obtain sufficiently low particle number concentrations. The AuNP reference suspension for the determination of the nebulization efficiency was diluted 10^6^-times to reach a mass concentration of 50 ng/L. The ionic calibration standards were used with concentrations between 100 ng/L and 1000 ng/L.

Between each measurement the complete system was rinsed in a two-step procedure using a 1% HCl + 1% HNO_3_ + 0.1% TritonX100 mixture and UPW to remove all potential particulate residues from the previous measurement and to avoid other memory effects, which was monitored by regular analysis of blank samples [ISO/TS 19590].

Data evaluation was performed with the MassHunter software (Version 4.6, Agilent Technologies, Waldbronn, Germany) using the automatic particle integration mode. The differentiation between particulate and ionic concentration was specified manually and set to the first minimum of the frequency-intensity diagram [71]. The construction of the analyte and reference material calibration response lines was conducted in Excel (Microsoft Office 2010, Version 14, Microsoft Corporation, Redmond, WA, USA), and the obtained values were transferred to the spICP-MS evaluation software to use a more meaningful calibration procedure. Measurements of mNP samples with results below the lower particle concentration limit of detection (LOD_conc_) were set to zero. A particle number of 10 particles over the complete acquisition time was considered equal to the concentration detection limit LOD_conc_, which corresponds to a particle concentration of around 4.98 × 10^5^ particles/L [72]. The size detection limit LOD_size_ was determined to around 15 nm for AgNP and AuNP, respectively. All investigated particle sizes were considerably above this limit with the exception of AgNP. Ionic concentrations above 150 ng/L for Au and 100 µg/L for Ag, respectively, were significantly above the detection limit and were taken into consideration. Thereby the aforementioned values were already corrected with the applied dilution factor.

### 2.3. Determination of Size Distribution, Particle Shape and Thickness of Protein Corona via Transmission Electron Microscope (TEM)

Pioloform-coated copper grids (G2440C) from Plano (Wetzlar, Germany) were incubated with 0.1% poly-l-lysine at 23 °C for 30 min, rinsed with water, and dried under dust-free atmosphere. A droplet of 20 µL of the sample was pipetted on the grids. After 20 min, the grids were rinsed with water and left for drying. Afterwards, the samples were directly investigated in the transmission electron microscope (TEM). TEM images were acquired with a Philips CM10 instrument, coupled with a CCD camera (IDS, Obersulm, Germany), at an acceleration voltage of 80 kV.

For visualization and measurement of the mNP protein corona thickness, the samples must be negatively stained. Therefore, the samples were treated with a saturated ethanolic uranyl acetate solution for 2 min. Excess liquid was removed, and the samples were dried again before TEM analysis. Particle dimension and thickness of the protein corona were evaluated using the software ImageJ [73], and the size distribution of 45 individual particles was analyzed. For determining the thickness of the protein corona 20 individual particles were analyzed.

### 2.4. Determination of Surface Charge via Electrical Asymmetrical Flow Field-Flow Fractionation Coupled to an UV–VIS-Detector (EAF4-UV-VIS)

UPW from a Milli-Q system (Integral 5 system, Merck, Darmstadt, Germany) was filtered through a 0.1 µm pore size membrane (Durapore, Merck Millipore, Tullagreen, Ireland). An optimum carrier solution for all samples consisted of a 0.4 mM Na_2_CO_3_ (Merck, Darmstadt, Germany) solution with a conductivity of around 90 µS/cm. The mNP were diluted to 10 ppm in UPW and CCM, respectively.

After incubation in CCM for at least 2 h the samples were analyzed by electrical asymmetrical flow field-flow fractionation (EAF4). The EAF4 fractionation system (EAF2000 MT, Postnova Analytics, Landsberg a. Lech, Germany) was equipped with an autosampler (PN5300), Slot Outlet (PN1650), channel thermostat (PN4020) and an Electrical FFF Module (PN2410), which controlled and applied the electrical field, as well as monitored the conductivity of the carrier solution. A regenerated cellulose membrane of 10 kDa molecular weight cut-off and a 350 µm height Mylar spacer were placed inside an electrical analytical fractionation channel with a tip-to-tip length of 277 mm. The temperature of the channel thermostat was kept constant at 25 °C while the samples were stored in the autosampler at 4 °C. A UV–VIS detector (PN3211) was coupled online to the EAF4 system. Due to differences between the UV absorbances of the investigated mNP, the absorbance wavelength of the UV–VIS detector was set to 515 nm (AuNRods), 526 nm (AuNP-30), 580 nm (AuNP-200) and 400 nm (AgNP), respectively, in order to obtain sufficient signal heights. The instrument was controlled by the NovaFFF software (Version 2.1.0.4, Postnova Analytics, Landsberg a. Lech, Germany).

The EAF4 fractionation method was adjusted to the respective mNP to retain sufficient fractionation. The detector flow rate and slot flow rate were both kept constant at 0.30 mL/min and 0.20 mL/min for all fractionations, respectively. Furthermore, an injection flow rate of 0.20 mL/min, an injection time of 6 min and a transition time of 0.2 min were used. The initial cross flow rate ranged from between 0.80 mL/min and 1.00 mL/min for all AuNP experiments and 1.20 mL/min for the AgNP fractionations. The individual cross flow profiles are displayed in Table S1.

A series of EAF4 fractionations with varying electrical field strengths (between 0 V/m and + 10 V/m) were performed, while keeping all other separation parameters constant. The electrophoretic mobility was determined from the retention time shift of the peak maximum under different electrical field strengths. A detailed description of the procedure is described elsewhere [74]. The conversion from electrophoretic mobility to the ζ-potential was conducted using the Helmholtz–Smoluchowski equation and Smoluchowski approximation with the Henry’s function f(κa) = 1.5, where κ represents the inverse Debye length and a the particle radius [75]. The data evaluation was performed with the NovaAnalysis software (Version 2102, Postnova Analytics, Landsberg a. Lech, Germany).

### 2.5. Cell Lines and Cultivation

Caco-2, a human adenocarcinoma cell line with epithelial morphology, and THP-1, a human monocytic cell line derived from an acute monocytic leukemia patient, were obtained from DSMZ (Deutsche Sammlung für Mikroorganismen und Zellkulturen GmbH, Braunschweig, Germany). HT29-MTX-E12, a mucus-secreting subclone from colon adenocarcinoma HT29 cells, differentiated into mature goblet cells by methotrexate, was obtained from Sigma-Aldrich (product no. 12040401, Munich, Germany).

All cell lines were cultured in Dulbecco’s Modified Eagle’s Medium (DMEM) high glucose (4.5 g/L) (Invitrogen, city, Germany) supplemented with 10% fetal calve serum (FCS) (Invitrogen), 2 mM l-Glutamine (Invitrogen), 1% penicillin/streptomycin (Invitrogen) and 1% non-essential amino acids (Invitrogen). All cell lines were cultured in a humidified incubator at 37 °C and 5% CO_2_ and passaged twice a week.

Then, 24 h prior to the seeding for the translocation studies, THP-1 cells were differentiated to adherent macrophage-like cells at a cell density of 4.0 × 10^5^ cells/mL in CCM supplemented with 20 ng/mL PMA (Phorbol-12-myristat-13-acetat, Sigma Aldrich, St. Louis, MO, USA).

### 2.6. Translocation Studies

For the in vitro GI translocation studies, 21-day differentiated triple co-cultures consisting of Caco-2, HT29-MTX-E12 and THP-1 were used in Transwell^®^ inserts with a pore size of 3.0 µm and a growth area of 1.12 cm^2^ (Corning^®^, New York, NY, USA).

2.5 × 10^5^ differentiated THP-1 cells were seeded basolaterally on flipped Transwell^®^ inserts and cultured for 1 h at 37 °C. Caco-2 and HT29-MTX were added at a density of 1.0 × 10^5^ per insert (ratio 9:1) apically (on the back flipped inserts) and cultured in a humidified incubator at 37 °C and 5% CO_2_ for 21 days to allow the formation of a dense barrier and enable the differentiation of the involved cell types [76,77]. CCM was exchanged every second day (apical volume 0.5 mL, basolateral volume 1.5 mL).

Before in vitro exposure the mNP test solutions (Section 2.1) were vortexed (30 s). Afterwards the mNP were pipetted apically (750 µL) to the triple co-culture model. On the basolateral side 1.5 mL NP-free CCM was added. The cells were exposed at 37 °C for 10 min, 30 min, 1 h and 4 h, in the case of AuNRods, AuNP-30 and AuNP-200 and for 1 h and 24 h in the case of AgNP. At these individual time points medium samples of 500 µL were collected on the apical and basolateral side in an Eppendorf tube (Eppendorf, Hamburg, Germany) and analyzed by spICP-MS and EAF4-UV–VIS. Each individual time point was operated with a separate cell culture insert. Empty cell culture inserts without cells served as blank controls. Translocated fraction, defined as the fraction of AuNP and AgNP that were measured in the basolateral medium and the amount of applied AuNP and AgNP to the apical chamber, was calculated from the results obtained by spICP-MS.

After collecting the medium samples for mNP characterization and analysis regarding translocation across the cell barrier, the in vitro model was used for cell viability and gene expression studies. In parallel, the cell models were analyzed via electron microscopy regarding NP uptake and accumulation.

### 2.7. Characterization of the In Vitro Model via Electron Microscopy

For light and electron microscopy investigation of the non-exposed and the exposed in vitro model, the cells were chemically fixed, dehydrated, embedded in resin and sectioned as described by Hesler et al. [76]. Staining of the sections was carried out with Löfflers Methylen blue solution (Carl Roth, Karlsruhe, Germany) followed by the investigation of the samples with a light microscope (Leica DM-LS, Leica, Wetzlar, Germany) [76].

For scanning electron microscopy (SEM), the fixed and dehydrated cells were dried by critical point drying using a CPD 010 (Baltec, Liechtenstein). After drying, the samples were mounted on aluminum stubs (Plano Wetzlar, Germany) and coated with gold using a sputter coater SCU 030 (Baltec, Liechtenstein). The investigations with the SEM DSM 940 (Zeiss, Oberkochem, Germany) were performed with 10 kV and the images were captured using the software DISS 5 (point electronic, Halle, Germany).

### 2.8. Cell Viability Assay

After 2 h and 24 h incubation with AuNRods, AuNP-30, AuNP-200 and AgNP, the cell viability of the triple co-culture GI model was determined using the alamarBlue^®^ assay, according to the manufacturer’s instructions. The supernatant on the apical side of the in vitro model was aspirated and replaced by 10% alamarBlue^®^ cell viability reagent in CCM (750 µL). 1% TritonX100 in CCM was applied as cell damaging positive control. The cells were incubated for 1 h at 37 °C. Afterwards, 100 µL of the apical supernatant was transferred from each cell culture insert into a minimum of three wells of a black 96well plate (Greiner Bio-One, Frickenhausen, Germany) for fluorescence measurement using a Tecan Infinite F200 plate reader (Tecan, Maennedorf, Switzerland) at an excitation/emission wavelength of 560/610 nm. Data evaluation was performed on Tecan i-control software (Version 1.9.17.0, Tecan, Maennedorf, Switzerland). The data calculation was performed in Excel (Microsoft Office 2016).

### 2.9. Determination of the Generation of Reactive Oxygen Species (ROS) via Quantitative Polymerase Chain Reaction (PCR)

For gene expression analysis of reactive oxygen species (ROS) associated genes, apical cultured cells of the triple co-culture (Caco-2 and HT29-MTX-E12) were harvested after 2 h and 24 h exposure. The RNA was isolated using the RNeasy Micro Kit (product code 74004, Qiagen, Hilden, Germany) according to the manufacturer’s instructions. cDNA was generated by reverse transcription of up to 500 ng RNA using the High-Capacity cDNA Reverse Transcription Kit (product code 4368814, Applied Biosystems^®^, Foster City, CA, USA) according to the manufacturer’s instructions. Gene expression was measured via the 5′-nuclease assay (TaqMan™) quantitative PCR (qPCR) of 2.5 ng DNA using a QuantStudio™ 7 Flex system with QuantStudio™ RealTime PCR-software (Version 1.3, Applied Biosystems^®^, Foster City, CA, USA) and TaqMan™ assays CAT (Assay ID Hs00156308_m1) and GPX1 (Assay ID Hs02516751_s1). Relative quantification was calculated with the 2^−^^∆∆CT^ method using HPRT1 (Assay ID Hs99999909_m1) as endogenous reference for normalization. The level of expression was evaluated in comparison with the chosen housekeeping gene HPRT1. The data calculation was performed in Excel (Microsoft Office 2010, Excel 2016).

### 2.10. Mathematical Characterization of Particle Translocation

Disposition of AuNP were characterized with a three-compartment modeling approach. Here, the compartments represent the apical compartment, cellular compartment and basolateral compartment, respectively. The cellular compartment not only represents the intracellular space but also the intermediate filter membrane between apical and basolateral cell arrangements. The following system of ordinary differential equation describes the change in fraction of applied particles in the respective compartment:

(1) $$\frac{dA_{a}\left(t\right)}{dt}=-k_{12}\times A_{a}\left(t\right)+k_{21}\times A_{c}\left(t\right)$$

(2) $$\frac{dA_{c}\left(t\right)}{dt}=k_{12}\times A_{a}\left(t\right)-k_{21}\times A_{c}\left(t\right)-k_{23}\times A_{c}\left(t\right)+k_{32}\times A_{b}\left(t\right)$$

(3) $$\;\;\frac{dA_{b}\left(t\right)}{dt}=k_{23}\times A_{c}\left(t\right)-k_{32}\times A_{b}\left(t\right)$$

(4) $$with\;A_{a}\left(t0\right)=\varphi +\eta_{i}{}_{\;}$$

where $A_{a},\;A_{c}$ and $A_{b}$ represent the fraction of applied particles present at time t in the apical, cellular and basolateral compartment, respectively, and $k_{12}$, $k_{21}$, $k_{23}$, $k_{32}\;$ are transfer constants.

The fraction available for translocation through the cell layers (*A_a_*(*t*0)) was estimated during the model parameter estimation step. Here, $\eta_{i}$ represents the normally distributed between-particle-species variability around the population mean φ. Further between-particle-species variability on all transfer constants was tested during model development while parameters were assumed to be log-normally distributed. For non-linear mixed effect modeling parameter estimation, a first-order conditional estimation method with interaction (FOCE-I) was used. Residual variability was implemented with a combined proportional and additive residual error model:

(5) $$Y_{ij}=A_{ij}\times \left(1+\varepsilon_{1ij}\right)+\varepsilon_{2ij}$$

where $Y_{ij}$ is the *j*th observed fraction for the *i*th nanoparticle species present in the apical or basolateral compartment, $A_{ij}$ is the corresponding model estimated fraction of applied particles, and $\varepsilon_{1ij}$ and $\varepsilon_{2ij}$ are the residual errors for the proportional and the additive components of the model with means of zero and variances of $\sigma_{1}^{2}$ and $\sigma_{2}^{2}$, respectively (i.e., $\varepsilon \;\sim \;N\left[0,\sigma^{2}\right]$).

For mathematical modeling and simulation, NONMEM^®^ version 7.4 (Icon Development Solutions, Ellicott City, MD, USA) and the R package mrgSolve [78] were used. Data management, statistical analyses and generation of graphics were performed with the R programming language version 4.0.2 (R Foundation for Statistical Computing, Vienna, Austria). Stochastic model simulations to estimate 95% confidence (CI) and 68% prediction intervals (PI) were performed with n = 2000 replicates. For each AuNP species, both simulated mean, CIs and PIs of translocated and non-translocated fractions including observed data were plotted against time. Upper and lower limits of PIs were post-processed by applying a Savitzky–Golay filter (polynomial degree of two and α of 0.75).

### 2.11. Statistical Analysis

Results of the physicochemical characterization and the toxicology studies are presented as mean with standard error of the mean of 3 independent experiments (n = 3), unless otherwise mentioned. Effects were compared to non-treated cells, and statistical analysis by Welch´s t-test was performed in Excel (Microsoft Office 2010, Excel 2016). The *p*-values are marked by * as *p* < 0.05, ** as *p* < 0.01 and *** as *p* < 0.001.

In order to test for differences in fraction of applied particles present in the apical and basolateral compartment, respectively, between AuNRods, AuNP-30 and AuNP-200, a one-way analysis of variance (ANOVA) was performed for each investigated time point (10, 30, 60 and 240 min) with a significance level α of 0.05.

## 3. Results and Discussion

### 3.1. Nanomaterial Characterization

#### 3.1.1. Size and Shape Analysis of the Nanoparticles via Transmission Electron Microscopy (TEM)

**Figure 1:**
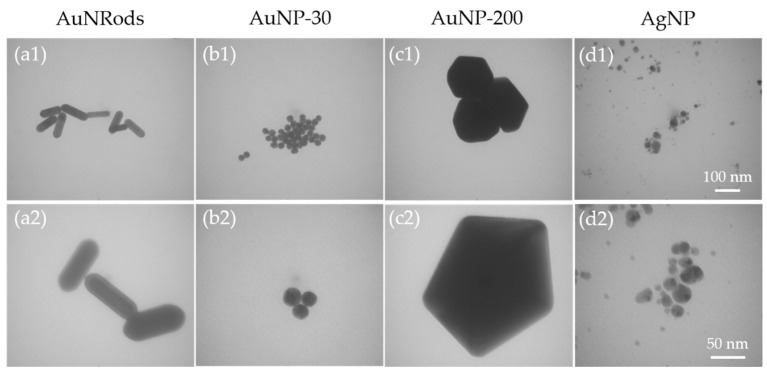
Representative transmission electron microscopy (TEM) images of metallic nanoparticles (mNP) in pure water. (**a1**,**a2**) gold rods (AuRods); (**b1**,**b2**) spherical 30 nm gold nanoparticles (AuNP-30); (**c1**,**c2**) spherical 200 nm gold nanoparticles (AuNP-200); (**d1**,**d2**) spherical 20 nm silver nanoparticles (AgNP). Scale bar in upper row: 100 nm. Scale bar in lower row: 50 nm.

TEM images of the gold nanorods (AuNRods) verify the typical rod-shaped structure, with a length of 120.8 nm ± 17.9 nm, a diameter of 36.0 nm ± 5.7 nm and an aspect ratio of about 3.2 (Figure 1(a1,a2)). Via TEM analysis, the 30 nm gold nanoparticles (AuNP-30) show a predominantly round shape with only a small amount of angular shaped particles and a mean diameter of 24.0 nm ± 2.2 nm (Figure 1(b1,b2)). The 200 nm gold nanoparticles (AuNP-200) present predominantly an angular shape with only a small amount of round particles. They have a mean diameter of 214 nm ± 20.1 nm (Figure 1(c1,c2)). Analysis of the Ag nanoparticles (AgNP) verifies the round-shaped form and a variety in diameter from 9 nm to 31 nm, with a mean diameter of 15.2 nm ± 7.1 nm (Figure 1(d1,d2)). Most of the particles are round shaped, and they have an extreme tendency to agglomerate.

#### 3.1.2. Stability Analysis in Cell Culture Medium (CCM)

After physicochemical characterization of the mNP in the stock suspension, the stability of AuNRods, AuNPs and AgNPs in CCM was determined by analyzing the samples after incubation in CCM. Additionally, the mNP, as received from the manufacturer, were characterized after dilution only with UPW (Figures S1–S8). Thereby, no significant deviations from the native size distributions and the size distributions after incubation in CCM were obtained for AuNRods and AuNPs using spICP-MS and TEM analysis (Table 1). The AgNP showed a broader size distribution with the lowest sizes overlapping with the LOD_size_ of the spICP-MS, which made it difficult to clearly distinguish particles from the ionic background. As a consequence, smaller particles (<LOD_size_) were counted as ions and therefore contributed to the ionic concentration. All results revealed meaningful and low deviations. A deviation of around 8% between incubated samples and the sample suspended in UPW was observed for the AuNP-200 sample, which was still considered as insignificant, whereas excellent comparability for the other mNP was obtained.

#### 3.1.3. Protein Corona Formation in Cell Culture Medium (CCM)

**Figure 2:**
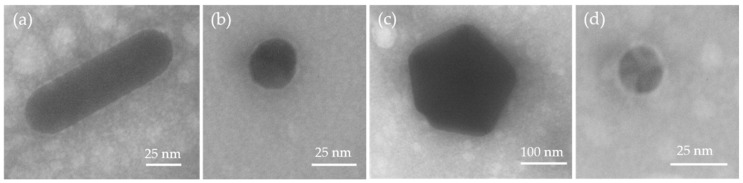
Representative transmission electron microscopy (TEM) images of metallic nanoparticles (mNP) with protein corona. The mNP were incubated in FCS-containing cell culture media and analyzed via TEM. (**a**) gold rods (AuNRods); (**b**) spherical 30 nm gold nanoparticles (AuNP-30); (**c**) spherical 200 nm gold nanoparticles (AuNP-200); (**d**) spherical 20 nm silver nanoparticles (AgNP).

After incubation in FCS-supplemented CCM, all studied mNP formed a protein corona (Figure 2). The corona is clearly visible via TEM, and its formation is independent of the particle size and shape.

#### 3.1.4. Determination of the Surface Charge by EAF4-UV–VIS

EAF4 allows a size-resolved determination of the electrophoretic mobility, respectively ζ-potential, by applying a cross flow field and a superimposed electrical field. The electrophoretic mobility values and ζ-potential values were evaluated for the UV–VIS-peak maximum of the fractionated mNP. The surface charge of the incubated samples was directly compared with the calculated values obtained for mNP from the stock suspensions that were diluted in UPW (Table 2, Figures S5–S8). In general, all results indicated an increasing ζ-potential value after suspension in CCM with only the AgNP samples revealing a significant drop in ζ-potential. The determined ζ-potential of AuNP-200 increased by more than 20%, while concurrently, the variation for AuNP-30 and AuNRods was insignificant and within the range of uncertainty with less than 10%. Especially for AuNP-30 in CCM a meaningful retention time shift was observed, which may correlate with an increased hydrodynamic size that may result from the formation of a protein corona (Figure S5).

Furthermore, AgNP also yielded an increased retention time but a broadened UV–VIS-peak by EAF4 fractionation, in contrast to the native AgNP was detected, which can be explained by a certain degree of agglomeration by the presence of CCM components (Figures S4 and S6). The distinct formation of a protein corona for AuNP-30 and AgNP was described in the previous section and was supported by the TEM analysis (Figure 2).

The decreasing tendency of the ζ-potential for AgNP may result from different stabilizers and stabilization mechanisms, respectively, compared to the AuNP samples. That may cause differences in the formed protein corona or may be related to the adsorption of different CCM components. The AgNP are stabilized both sterically and electrostatically [79]. However, the shifted retention time maximum and especially the broadened size distribution after incubation in CCM made it difficult to compare both ζ-potential values as the fractograms are not fully comparable. In general, increased retention times in EAF4 (with no electrical field) correspond to an increased hydrodynamic size, but it should be mentioned that changed surface properties (such as zeta potential) may cause a different retention behavior in EAF4 due to particle-membrane interactions.

### 3.2. Characterization of the In Vitro Model by Light and Electron Microscopy

**Figure 3:**
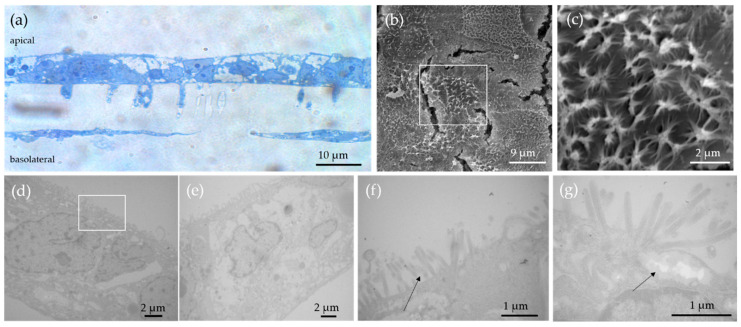
TEM images of the gastrointestinal in vitro model. The different intestinal cell types of the triple co-culture (Caco-2 cells, HT29-MTX-E12 cells and THP-1 cells) after 21 days of cultivation could be identified. (**a**) Light microscopic images of HE stained GI co-triple-culture. Caco-2 and HT29-MTX-E12 cells are cultured on the apical side of the membrane of the cell culture insert, THP-1 cells on the basolateral side. (**b**) SEM image of the surface of the GI triple co-culture. (**c**) Enlargement of the SEM image marked in (**b**) showing the microvilli of the Caco-2 cells. (**d**,**e**) TEM image of the GI triple co-culture. (**f**) TEM image of the GI triple co-culture. Black arrows indicate the microvilli. (**g**) TEM image of the GI triple co-culture. Black arrows indicate the encapsulated mucus which is secreted by the HT29-MTX-E12 cells.

Before performing the translocation studies, the developed triple in vitro culture was characterized by different microscopy techniques. Light microscopy provides an overview of the structure and allocation of apical and basolateral cell layers as well as the distribution of the different cell types. Subsequently, these are imaged in relation to the surface structures in the SEM and analyzed in relation to the internal structures in the TEM. The investigation of the semi-thin sections via light microscope, after hematoxylin and eosin staining, shows layers of two different cell types at the apical side of the sample and a mostly confluent cell monolayer at the basolateral side of the culture (Figure 3a). SEM analysis of the triple in vitro model shows a multilayer of cells with different topographical appearance on the apical side of the membrane (Figure 3b,c). Cells with a large number of microvilli alternate with cells with few or no microvilli on their surface (Figure 3c). On the surface of some cells, the microvilli are found in small clusters (Figure 3c). Via TEM, we could differentiate between three various cell types. The typical form of the Caco-2 cells (Figure 3d–e), consisting of a significant quantity of microvilli on the surface, was identified as well as the mucus secreting HT29-MTX-E12 cells which show a less dense cytoplasm. At higher magnification, the mucus vesicles at the cell surface are also visible in addition to the microvilli at the cell surface (Figure 3f–g). The individual borders between the two different cell types are also clearly visible in Figure 3g. Both cell types are separated by a cell membrane border and some connecting desmosomes.

### 3.3. Gastrointestinal Translocation of mNPs

**Figure 4:**
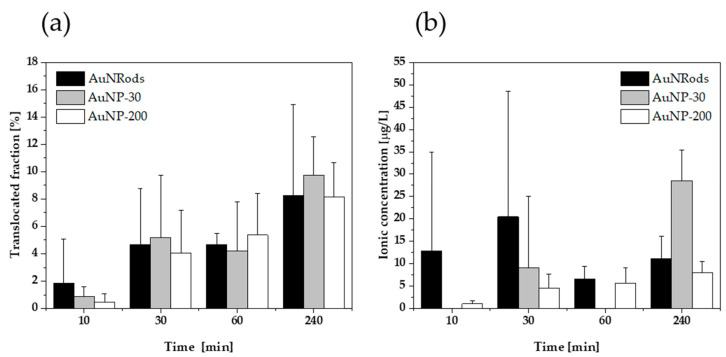
Gastrointestinal translocation of AuNRods and AuNPs determined by spICP-MS. (**a**) Relative particulate basolateral concentrations of the investigated AuRods and AuNPs in dependence of time. (**b**) Basolateral ionic concentration in dependence of time. n = 3.

The triple co-culture model was exposed with the mNPs for different durations (10 min, 30 min, 1 h and 4 h). After the individual time points, the medium samples in the apical and basolateral compartment were analyzed via spICP-MS. An increase in translocated particle concentration over time from around 0.4 ± 0.6% up to 9.8 ± 2.8% was observed for the studied AuNMs (Figure 4a). Hereby, the determined basolateral particulate concentrations were correlated to the apical inserted particle concentration and displayed in percent as translocated fraction. Particle number concentrations of 9.22 × 10^9^ particles/L (AuNP-200), 6.91 × 10^9^ particles/L (AuNRods) and 3.80 × 10^12^ particles/L (AuNP-30), respectively, were introduced on the apical side at the start time. Figure 4a visualizes the time-dependent relationship of the basolateral particle concentration. No significant size or shape dependence on the translocated concentration was determined, when comparing AuNP-30 with 9.8 ± 2.8% to 8.2 ± 2.6% of AuNP-200 nm and 8.2 ± 6.6% AuNRods, respectively.

In parallel, the ionic concentration increased with time, showing similar trends for all particle sizes and shapes. The ionic concentration on the basolateral side by the application of a cell barrier ranged between 0.00 µg/L and 28.5 µg/L ± 6.9 µg/L (Figure 4b). Throughout all measurement series, the respective blank samples that only contained CCM showed the absence of particles with particle number concentrations below the LOD_conc_. The necessity of high dilutions to obtain low particle concentrations together with extremely low particle mass concentration limits resulted in relatively high ionic concentration detection limits due to the fact that the background gets diluted by the same factor [80]. However, the results indicate low ionic concentrations with a small number of samples below the ionic concentration detection limit.

All results of the AuNM experiments were transferable to the experiments with AgNPs. For AgNP studies, the triple co-culture model was exposed for 2 h and 24 h. A particle number concentration of 2.70 × 10^14^ particles/L was inserted. After 2 h in the basolateral compartment, the translocated fraction of AgNPs was 3.0 ± 1.8%. After an incubation time of 24 h, a translocated fraction of 14.0 ± 5.2% and an ionic basolateral concentration of 2754 µg/L ± 4771 µg/L were detected in contrast to ionic concentrations below the limit of detection after 2 h. Dissolution and reactivity of AgNP in biological media have been reported in literature [70,81]. Since AgNPs might dissolve in the medium, the ionic concentration of silver was also determined in the apical samples. Over the investigated time period no significant dissolution and increase in ionic concentration of AgNPs could be determined by sp ICP-MS (data not shown). Components of the surrounding CCM may stabilize the particles and prevent dissolution [82,83].

As mentioned in the previous section, EAF4-UV–VIS indicated a shift towards larger sizes for AgNP in CCM (Figure S6). Moreover, spICP-MS revealed an increased fraction of larger particle sizes (Figure S4), which on the other hand had no significant influence on the median size average. Some results came along with large standard deviations, especially the experiments with AuNRods, which can be explained by variations in the inserted particle number concentration. The concentration of the prepared mNP-CCM that was inserted on the apical side was determined for each experiment and compared to the concentration, which was obtained for the different durations on the basolateral side. Therefore, the translocated fraction values take varying start concentrations throughout all replications (n = 3) into consideration.

### 3.4. Mathematical Characterization of Particle Translocation

**Figure 5:**
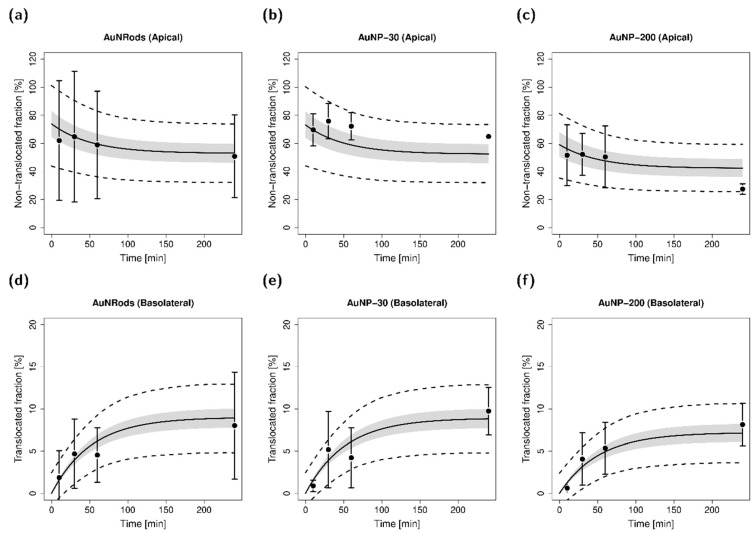
Disposition of AuNRods, AuNP-30 and AuNP-200 in the apical (**a**–**c**) and basolateral (**d**–**f**) compartment, respectively. Observed fractions of applied particles are depicted as circles (arithmetic mean and standard deviation). Model simulation means are shown as lines. Shaded areas depict the 95% confidence interval; dashed lines depict the upper and lower limit of the 68% prediction interval.

One-way ANOVA revealed no significant differences in observed fraction of applied particles between different AuNP species, both for the apical and basolateral compartment and for all investigated time points with adjusted p-values always greater than 0.1. Estimation of transfer kinetics with the help of a compartmental translocation model showed no differences between AuNP species with estimated between-particle-species variabilities approaching zero and high levels of η-shrinkage. As a result, the final model did not include between-particle-species variability on transfer parameters. Results of the model simulations for each investigated AuNP species are depicted in Figure 5. While Figure 5a–c show observed and simulated fractions for the different AuNP species present in the apical chamber, Figure 5d–f depict the corresponding fractions in the basolateral chamber. Model simulations are in good agreement with mean observed values with respective model parameters presented in Table S2.

The results are in agreement with studies of Bachler et al. where no significant differences in translocated fraction could be observed for in vitro monolayer models with alveolar type II epithelial cells between AuNPs of size 18 nm–80 nm for an investigated size range of 2 nm–80 nm [64]. In contrast, other studies found differences in cellular uptake for transferrin-coated AuNP with an optimal size of 50 nm [65] assuming a size preference for receptor-mediated endocytosis—the suggested primary route of cell entry [66]. Due to high variability in longitudinal data, combined with the complexity of the investigated triple co-culture cell model, cell entry via membrane wrapping of nanoparticles [67] was necessarily simplified to kinetics via transfer rate constants.

The mean fraction available for translocation (φ) of AuNP species was estimated to be 69% (AuNRods: 74%, AuNP-30: 73%, AuNP-200: 59%). It should be noted that the estimated fraction available for translocation comprises of different factors, such as incomplete AuNP recovery in the apical compartment or adhesion of AuNPs to the cell surface. In general, various influences such as cell line type used or applied AuNP dose could have affected cellular uptake and translocation. Although modeling could not identify differences in fractional cellular uptake and translocation kinetics for AuNPs, these factors and the observed high intra-species-variability might mask existing differences in translocation kinetics between AuNP species.

### 3.5. Cellular Uptake and Cell Viability

**Figure 6:**
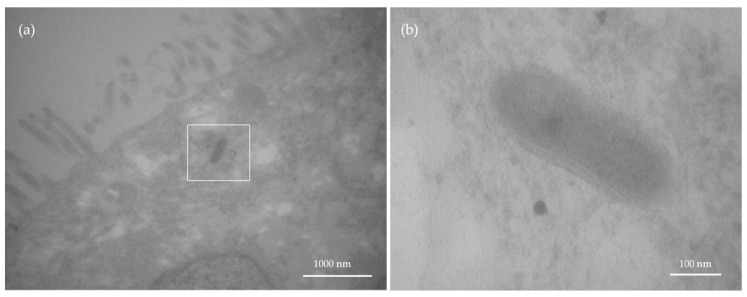
TEM image of an internalized AuNRod in the gastrointestinal in vitro model. After AuNRod exposure, the in vitro model was investigated by TEM. (**a**) Section through the cell model. (**b**) Enlargement of the area marked in (**a**). Clearly visible here is the membrane bilayer around the AuNRod, through the protein corona and the vesicle membrane.

After completing the translocation studies, the exposed cells were analyzed by TEM to study cellular uptake of the mNP. Using AuNRods as an example, cellular uptake was observed (Figure 6). AuNRods were found in the cytoplasm of the cells (Figure 6a). Detailed analysis showed that the AuNRods contain a second shell in addition to the protein corona (Figure 6b). This is most likely a vesicular membrane. This fact proves that the AuNRods are internalized by the cells of the GI in vitro model via endocytosis.

**Figure 7:**
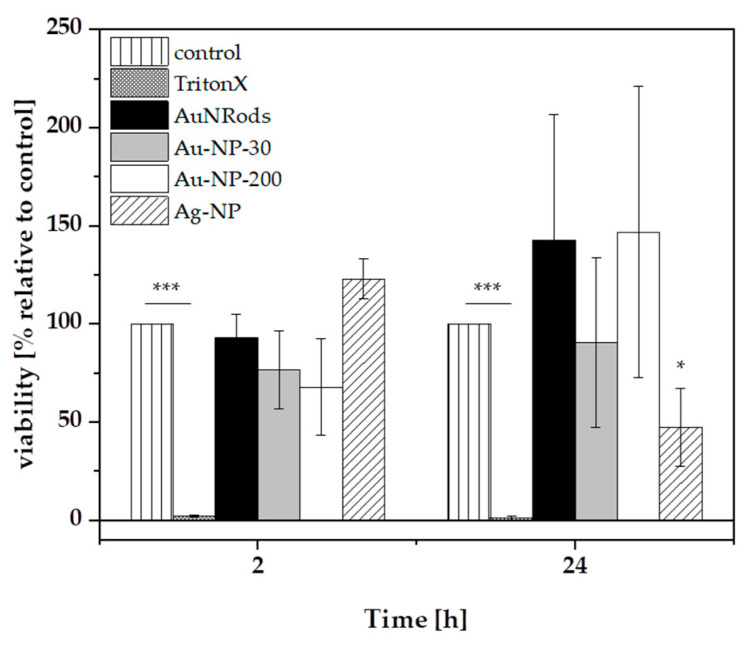
Cell viability of the gastrointestinal in vitro model after 2 h and 24 h incubation with mNPs. The in vitro model was exposed to 1 µg/mL AuNRods, AuNP-30, AuNP-200 and 30 µg/mL AgNP. Afterwards, the cell viability of the apical cell layer (Caco-2 and HT29-MTX) was analyzed via alamarBlue^®^ assay. n = 3, * *p* ≤ 0.5, *** *p* ≤ 0.001.

After the NP translocation experiments, the cell viability was determined after 2 h and 24 h. The positive control (1% TritonX treated in vitro model) decreased in viability to 2.22 ± 0.53% after 2 h and 0.99 ± 1.30% after 24 h, demonstrating the effectiveness of the cell death inducing detergent, as well as the functionality of the test method (Figure 7). Cells incubated with NP-free CCM were defined as negative control and set as 100% viability. After 2 h, none of the applied mNPs induced cell death in comparison to the negative control. After 24 h, AgNP decreased the cell viability significantly to 47.69 ± 1.11% (Figure 7). This decrease in cell viability might have influenced translocation kinetics of AgNP. After 24 h, an insignificant trend of an increased cell viability was observed in case of AuNRods and AuNP-200, which could be explained by a higher cell metabolism acting as a type of defense mechanism in response to the presence of foreign particles inside the cells. A recent study described a low cytotoxicity induced by AuNP (60 nm) in a Caco-2 monoculture after 24 h exposure. This monoculture was also differentiated for 21 days on membrane inserts before NP exposure, as in our study. Unlike our study, the AuNP were not able to cross the intestinal barrier [32]. In contrast to their findings, another study described the time-dependent translocation of AuNP in the sizes 15, 50 and 100 nm across Caco-2 monolayers, evidenced by ICP-MS measurements, similarly to our findings with the same sensitive method but in a triple co-culture model [55]. The cellular uptake and cytotoxicity of AuNP in the sizes 5, 50 and 100 nm were determined by Jiang et al., who did not observe any cytotoxic effects or disturbances of the epithelial barrier permeability regardless of the particle size [34]. The induction of acute cytotoxicity via an oxidative-stress-related pathway was observed for AgNP but not for AuNP in Caco-2 cells [84]. This corresponds to our finding that AgNP, but not AuNP or AuNRods, induces cytotoxicity in a triple co-culture model after 24 h, but not after 2 h. In a comparable triple co-culture model in the study of Susewind et al., Ag-NP also induced cytotoxicity after 24 h but not AuNP (15 nm and 80 nm). Furthermore, it could be demonstrated that the Caco-2 monoculture alone was more sensitive to the mNP than the triple co-culture model [56]. Thus, for more physiological relevant screenings of mNP, triple co-culture-models of the GI barrier should be applied, instead of Caco-2 monocultures.

### 3.6. Expression of Oxidative Stress Genes after mNP Exposure

**Figure 8:**
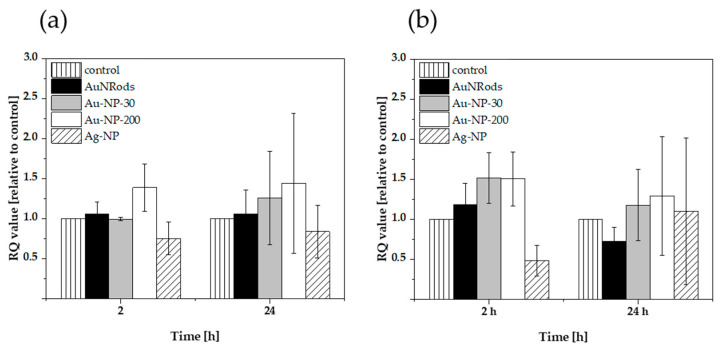
ROS generation in the gastrointestinal in vitro model after 2 h and 24 h incubation with mNPs. The in vitro model was exposed to 1 µg/mL AuNRods, AuNP-30, AuNP-200 and 30 µg/mL AgNP. Afterwards, the expression of Catalase (CAT) and Glutathioneperoxidase (GPX1) in the apical cells (Caco-2 and HT29-MTX) was determined via qPCR. (**a**) CAT expression; (**b**) GPX1 expression. n = 3.

After the mNP translocation experiments, the cells were harvested, RNA was extracted, and cDNA was produced to determine the gene expression of two ROS associated genes (CAT and GPX1) by qPCR. Catalase (CAT) acts as an enzyme catalyzing the reaction from hydrogen peroxide (H_2_O_2_) to water and oxygen, a defense mechanism against oxidative stress, mitigating the toxic effects of H_2_O_2._ The glutathione peroxidase 1 (GPX1) is one of the most important antioxidant enzymes in humans and functions in the detoxification of H_2_O_2,_ protecting cells against oxidative damage [85]_._ The possible ongoing production of H_2_O_2_ upon incubation with the tested mNP would require a higher activity of CAT and GPX1 in the cells; thus, the gene expression patterns would change in order to upregulate the protein synthesis of the needed enzymes. The gene expression patterns were examined after 2 h and 24 h mNP incubation (Figure 8). A double RQ-value (compared to the control) has been defined as threshold. A gene upregulation over this threshold is identified as effect in this study. None of the applied mNP induced a 2-times higher expression of the genes of interest at the investigated time points (Figure 8), neither for CAT enzyme as defense mechanism against oxidative stress (Figure 8a) nor for GPX1 antioxidant enzyme (Figure 8b). Thus, it can be assumed that both investigated ROS-mediated mechanisms were not active after 2 h and 24 h. One reason for that might be that the mechanisms are activated at an earlier or later time point or do not occur at all after exposure with the tested AuNRods, AuNP and AgNP in the GI triple co-culture-model. Moreover, the aspect of tested dose or cellular composition of the in vitro model could be a reason for the lack of gene upregulation. AgNP actually are known to induce cytotoxicity by ROS and free-radical generation, which can be traced back to the fact that AgNP release ions from their surface in aqueous solutions [47]. This effect can be reduced by different surface coatings [86]. Furthermore, AgNP have been described to decrease antioxidant enzymes and imbalance the oxidative status in the cells [86]. However, the two genes relevant for the ROS metabolism stayed unchanged after the incubation with uncoated AgNP in this study. AuNP with different sizes and coatings were also demonstrated in previous studies to induce oxidative stress in several cell types, such as HeLa cells [87], kidney cells [33], retinal cells [88] and MRC-5 fibroblasts [89]. In contrast to our study, Bajak et al. reported that small AuNP (5 nm) increased the glutathione metabolism in Caco-2 cells [90]. Besides the size, additional differences regarding incubation time, concentration, surface modification of the NPs, and investigating only one cell type in contrast to three could explain the different outcomes regarding the glutathione metabolism and oxidative stress mechanisms. In another study, it was demonstrated that smaller AuNP showed more adverse effects in vitro and in vivo [87,91]. The tested AuNP in this study were relatively large in comparison to others examined elsewhere, and the applied concentration of 1 µg/mL chosen was very low. In addition, the used model exhibits a mucous layer which can serve as a protectant layer from NPs [77]. These aspects may be the reason for non-toxic effects of the studied AuNP in different sizes.

## 4. Conclusions

In the present study, a combined approach of detailed physicochemical analysis, toxicological assays and in silico modeling was used to answer the question of the influence of the physicochemical characteristics and stability of AuNP on their translocation across the intestinal barrier and related biological effects. Using AuNP of different sizes and shapes (spheres with a mean diameter of 30 nm and 200 nm, rods with a mean diameter of 40 nm and a mean length of 112 nm) corona formation in cell culture medium, surface chemistry and further physicochemical parameters were correlated with their translocation properties and biological effects in an 3D in vitro GI barrier model, while AgNP were used for comparative purposes.

Both the physicochemical and biological analyses as well as the mathematical model did not detect any significant differences between the different particle species using the chosen study conditions. The combined approach used in the presented study may serve as an advanced testing strategy for an improved risk assessment of ENM in the future.

## Figures and Tables

**Table 1:** Size distribution of metallic nanoparticles (mNP). The different nanoparticles (gold nanorods (AuNRods), gold nanoparticles (AuNP) and silver nanoparticles (AgNP)) were analyzed regarding their size distribution in the stock solution as well as in cell culture media. Two techniques, spICP-MS and TEM, were used. For AuNRods an equivalent spherical size was calculated from the particle mass obtained by spICP-MS.

	spICP-MS Median Size (nm)	TEM Diameter (nm)	spICP-MS Median Size (nm)	TEM Diameter (nm)
	Stock Suspension		Cell Culture Medium	
AuNRods	57.4 ± 0.5	36.0 ± 5.7	54.5 ± 0.9	31.2 ± 7.5
AuNP-30	28.0 ± 0.1	24.0 ± 2.2	26.0 ± 1.0	25 ± 5.5
AuNP-200	191.6 ± 1.4	214 ± 20.1	176.2 ± 1.2	222 ± 18.7
AgNP	20.8 ± 0.2	15.2 ± 7.1	21.3 ± 0.5	15.5 ± 7.4

**Table 2:** ζ-potential results obtained from EAF4-UV–VIS analysis for different metallic nanoparticles (mNP). The electrophoretic mobility values for all investigated mNP and their respective ζ-potential values. The displayed uncertainties were obtained from the linear least squares analyses. UPW = ultrapure water, CCM = cell culture medium.

mNP	Solvent	Electrophoretic Mobility (1 × 10^−8^ m^2^/(V s))	ζ-Potential (mV)
AuNRods	UPW	−2.74 ± 0.22	−35.0 ± 2.8
AuNP-30		−4.61 ± 0.39	−59.0 ± 5.0
AuNP-200		−2.36 ± 0.10	−30.2 ± 1.2
AgNP		−2.99 ± 0.15	−38.2 ± 1.9
AuNRods	CCM	−2.68 ± 0.14	−34.2 ± 1.8
AuNP-30		−4.41 ± 0.32	−56.4 ± 4.1
AuNP-200		−1.81 ± 0.11	−23.1 ± 1.4
AgNP		−5.35 ± 0.35	−68.5 ± 4.5

## Supplementary Materials

The following are available online at https://www.mdpi.com/article/10.3390/nano11061358/s1. Table S1: EAF4 fractionation conditions for the different studied metallic nanoparticles (mNP). Table S2: Parameter estimates of gold nanoparticle translocation non-linear mixed effect modeling. Figure S1: Comparison of the native size distribution of AuNP-30 with the size distribution in CCM obtained by spICP-MS. Figure S2: Comparison of the native size distribution of AuNP-200 with the size distribution in CCM obtained by spICP-MS. Figure S3: Comparison of the native size distribution of AuNRods with the size distribution in CCM obtained by spICP-MS. Figure S4: Comparison of the native size distribution of AgNP with the size distribution in CCM obtained by spICP-MS. Figure S5: Fractogram with the UV/Vis signals of AuNP-30 comparing the sample in UPW and CCM. Figure S6: Fractogram with UV/Vis signals of AgNP comparing the sample in UPW and CCM. Figure S7: Fractogram with the UV/Vis signals of AuNP-200 comparing the sample in UPW and CCM. Figure S8: Fractogram with the UV/Vis signals of AuNRods comparing the sample in UPW and CCM.
