# Supplementary material for: Influence of Physicochemical Characteristics and Stability of Gold and Silver Nanoparticles on Biological Effects and Translocation across an Intestinal Barrier—A Case Study from In Vitro to In Silico
Source: Nanomaterials (Basel). 2021 May 21;11(6):1358. doi: 10.3390/nano11061358 (PMC8224057; doi:10.3390/nano11061358)
Supplement: Supplementary file 1 [file nanomaterials-11-01358-s001.zip › nanomaterials-1211917-supplementary.pdf]

nanomaterials

MDPI

Article

Influence of Physicochemical Characteristics and Stability of Gold and Silver Nanoparticles on Biological Effects and Translocation Across An Intestinal Barrier-A Case Study from in Vitro to in Silico

Yvonne Kohl [1,*], Michelle Hesler [1], Roland Drexel [2], Lukas Kovar [3], Stephan Dähnhardt-Pfeiffer [4], Dominik Selzer [3], Sylvia Wagner [1], Thorsten Lehr [3], Hagen von Briesen [1] and Florian Meier [2,*]

1 Fraunhofer Institute for Biomedical Engineering IBMT, Sulzbach, 66280, Germany; yvonne.kohl@ibmt.fraunhofer.de (Y.K.); michelle.hesler@ibmt.fraunhofer.de (M.H.); sylvia.wagner@ibmt.fraunhofer.de (S.W.); hagen.briesen@ibmt.fraunhofer.de (H.v.B.)
2 Postnova Analytics GmbH, Landsberg am Lech, 86899, Germany; roland.drexel@postnova.com (R.D.); florian.meier@postnova.com (F.M.)
3 Department of Clinical Pharmacy, Saarland University, Saarbrücken, 66123, Germany; lukas.kovar@uni-saarland.de (L.K.); dominik.selzer@uni-saarland.de (D.S.); thorsten.lehr@uni-saarland.de (T.L.)
4 Microscopy Services Dähnhardt GmbH, Flintbek, 24220, Germany; sd@msd.sh (S.D.-P.)
* Correspondence: yvonne.kohl@ibmt.fraunhofer.de (Y.K.); florian.meier@postnova.com (F.M.); Tel.: +49-6897-9071-256 (Y.K.); +49-8191-985-6880 (F.M.)

Citation: Kohl, Y.; Hesler, M.; Drexel, R.; Kovar, L.; Dähnhardt-Pfeiffer, S.; Selzer, D.; Wagner, S.; Lehr, T.; von Briesen, H.; Meier, F. Influence of Physicochemical Characteristics and Stability of Gold and Silver Nanoparticles on Biological Effects and Translocation Across An Intestinal Barrier-A Case Study from in Vitro to in Silico. *Nanomaterials* **2021**, *11*, 1358. https://doi.org/10.3390/nano11061358

Academic Editor: Ivana Vinkovic Vrcek

Received: 21 April 2021
Accepted: 13 May 2021
Published: 21 May 2021

Publisher's Note: MDPI stays neutral with regard to jurisdictional claims in published maps and institutional affiliations.

Copyright: © 2021 by the authors. Submitted for possible open access publication under the terms and conditions of the Creative Commons Attribution (CC BY) license (https://creativecommons.org/licenses/by/4.0/).

Table S1. EAF4 fractionation conditions for the different studied metallic nanoparticles (mNP).

**AuNP-30**	**Duration (min)**	**Cross flow rate (mL/min)**	**Cross flow decay type**
Elution step 1	5	1.00	Constant
Elution step 2	25	1.00	Linear
Elution step 3	3	0.25	Power, exp. 0.80
Elution step 4	7	0.18	Power, exp. 0.70
Elution step 5	5	0.10	Constant
Rinse step	10	0	-
**AuNP-200**	**Duration (min)**	**Cross flow rate (mL/min)**	**Cross flow decay type**
Elution step 1	5	0.80	Constant
Elution step 2	20	0.80	Power, exp. 0.10
Elution step 3	10	0.03	Constant
Rinse step	10	0	-
**AuNRods**	**Duration (min)**	**Cross flow rate (mL/min)**	**Cross flow decay type**
Elution step 1	5	1.00	Constant
Elution step 2	25	1.00	Power, exp. 0.20
Elution step 3	10	0.10	Constant
Rinse step	10	0	-
**AgNP**	**Duration (min)**	**Cross flow rate (mL/min)**	**Cross flow decay type**
Elution step 1	20	1.20	Constant
Elution step 2	15	1.20	Linear
Elution step 3	3	0.28	Power, exp. 0.80
Elution step 4	7	0.18	Power, exp. 0.70
Elution step 5	5	0.1	Constant
Rinse step	10	0	-

Table S2. Parameter estimates of gold nanoparticle translocation non-linear mixed effect modeling.

**Parameters**	**Estimate (% RSE)**	**Parameter description**
k_{12}	5.5×10^{-3} (35)	Transfer constant from apical to cellular compartment
k_{21}	2.4×10^{-2} (35)	Transfer constant from cellular to apical compartment
k_{23}	7.6×10^{1} (6)	Transfer constant from cellular to basolateral compartment
k_{32}	1.0×10^{2} (49)	Transfer constant from basolateral to cellular compartment
φ	6.9×10^{-1} (4)	Fraction available for translocation through the cell layers (population mean)
ω_{φ}^{2}	6.9×10^{-3} (93)	Between-particle-species variability on φ
σ_{1}^{2}	1.5×10^{-1} (48)	Variance of proportional residual error
σ_{2}^{2}	4.5 (36)	Variance of additive residual error

RSE: relative standard error; unit of transfer constants is in %/min

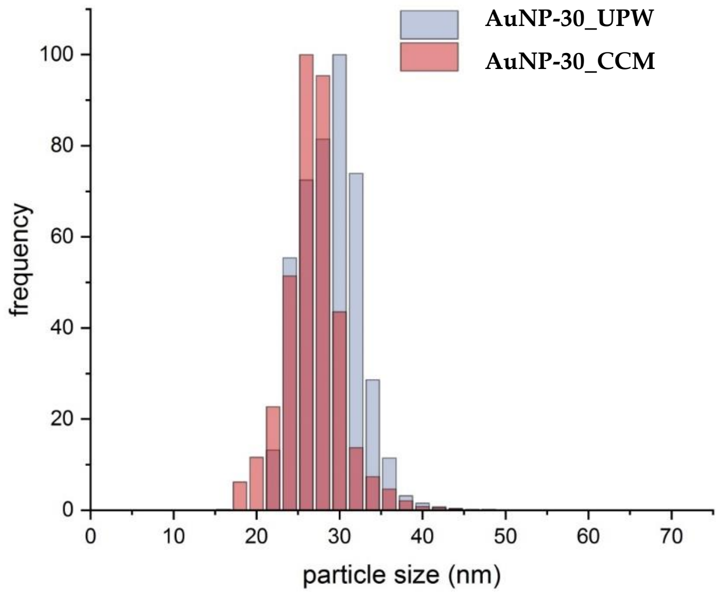

Figure S1. Comparison of the native size distribution of AuNP-30 with the size distribution in CCM obtained by spICP-MS. UPW = ultrapure water, CCM = cell culture medium.

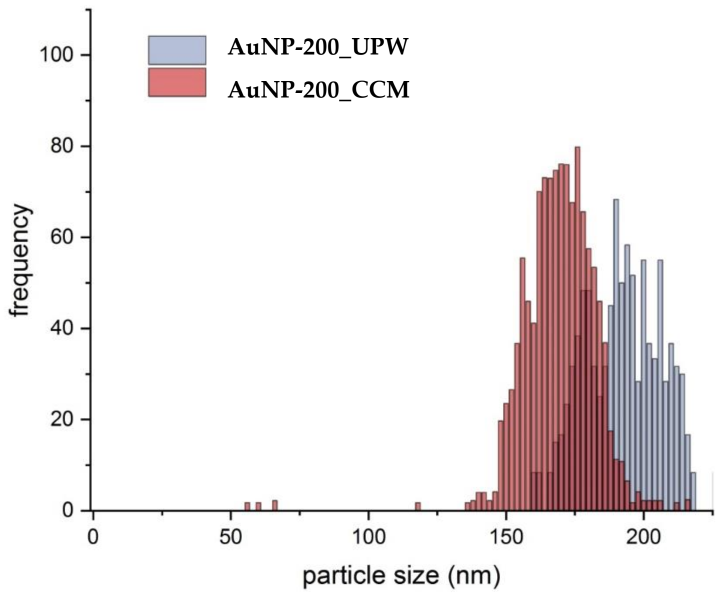

Figure S2. Comparison of the native size distribution of AuNP-200 with the size distribution in CCM obtained by spICP-MS. UPW = ultrapure water, CCM = cell culture medium.

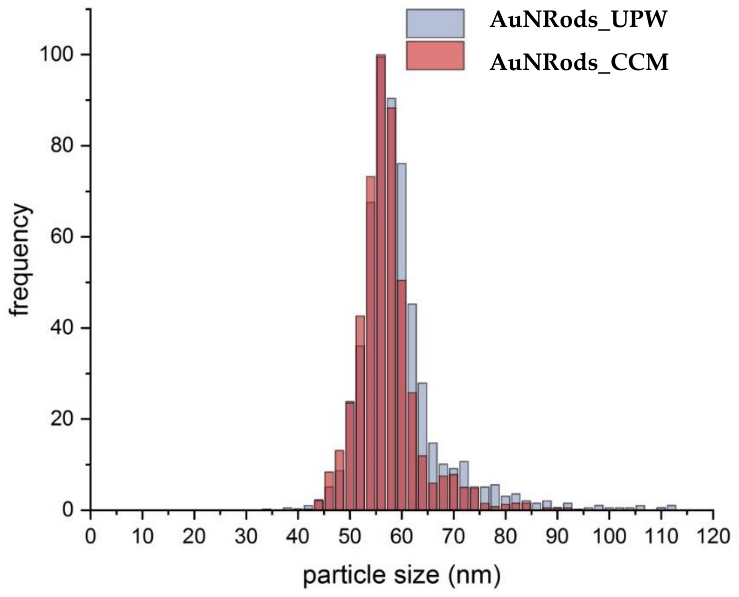

Figure S3. Comparison of the native size distribution of AuNRods with the size distribution in CCM obtained by spICP-MS. UPW = ultrapure water, CCM = cell culture medium.

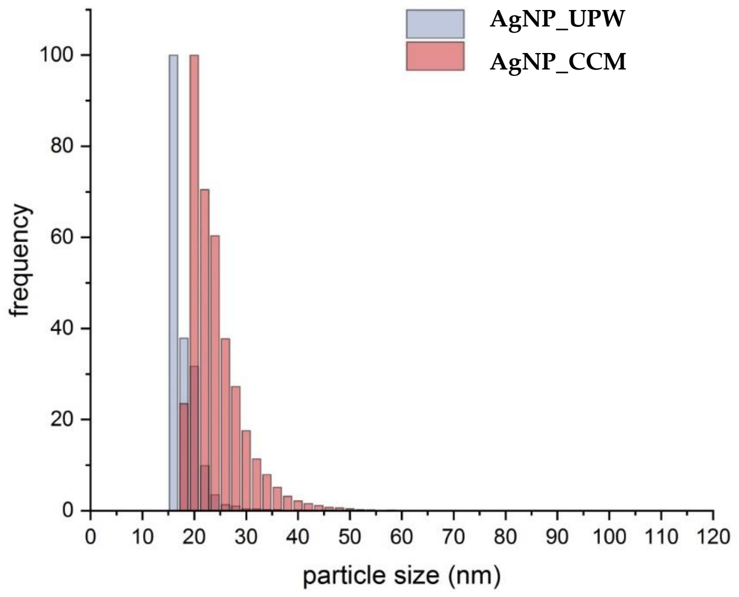

Figure S4. Comparison of the native size distribution of AgNP with the size distribution in CCM obtained by spICP-MS. UPW = ultrapure water, CCM = cell culture medium.

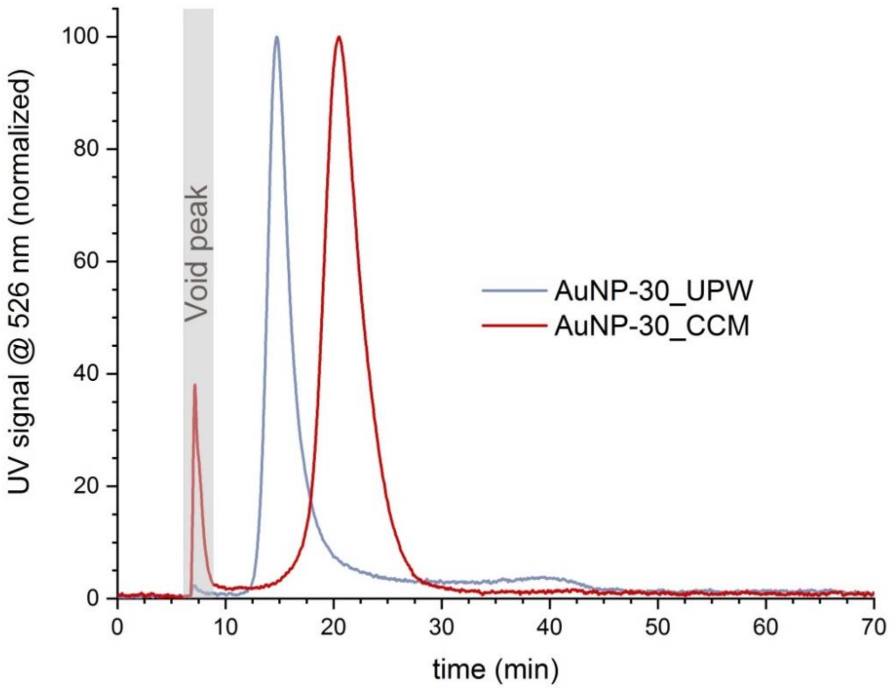

Figure S5. Fractogram with the UV/Vis signals of AuNP-30 comparing the sample in UPW and CCM. The size retention time was shifted to larger times, indicating an increased hydrodynamic size. UPW = ultrapure water, CCM = cell culture medium.

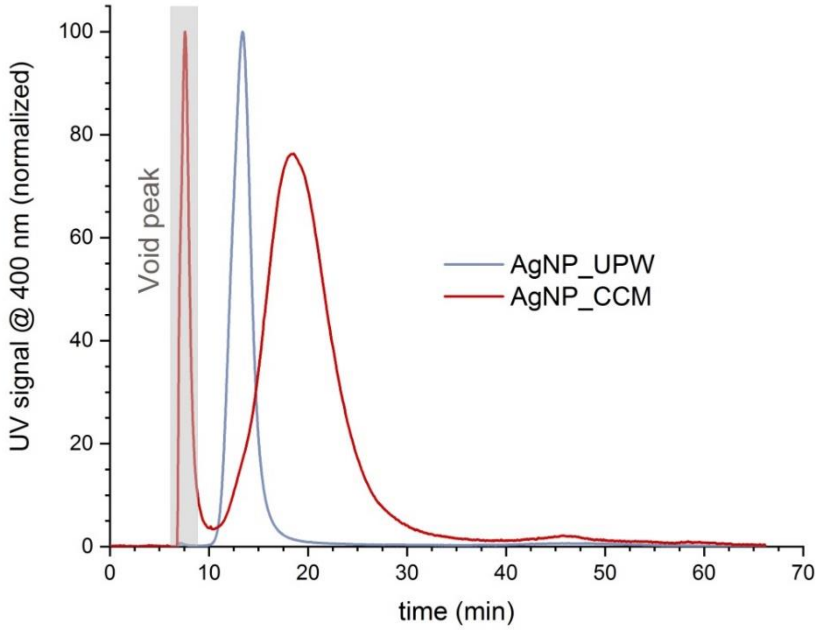

Figure S6. Fractogram with UV/Vis signals of AgNP comparing the sample in UPW and CCM. The retention time was shifted to larger times, indicating an increased hydrodynamic size, which came along with a significant broadening of the signal. The second observation suggests agglomeration. UPW = ultrapure water, CCM = cell culture medium.

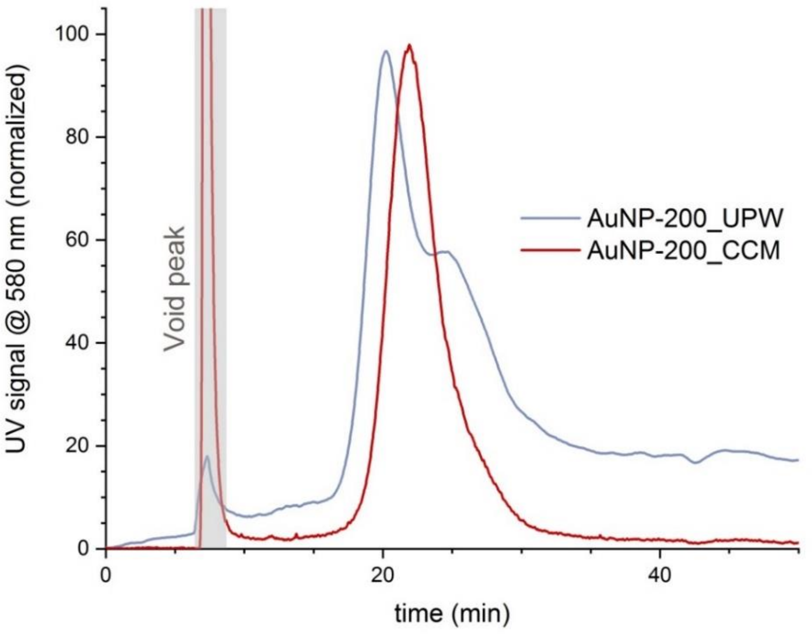

Figure S7. Fractogram with the UV/Vis signals of AuNP-200 comparing the sample in UPW and CCM. UPW = ultrapure water, CCM = cell culture medium.

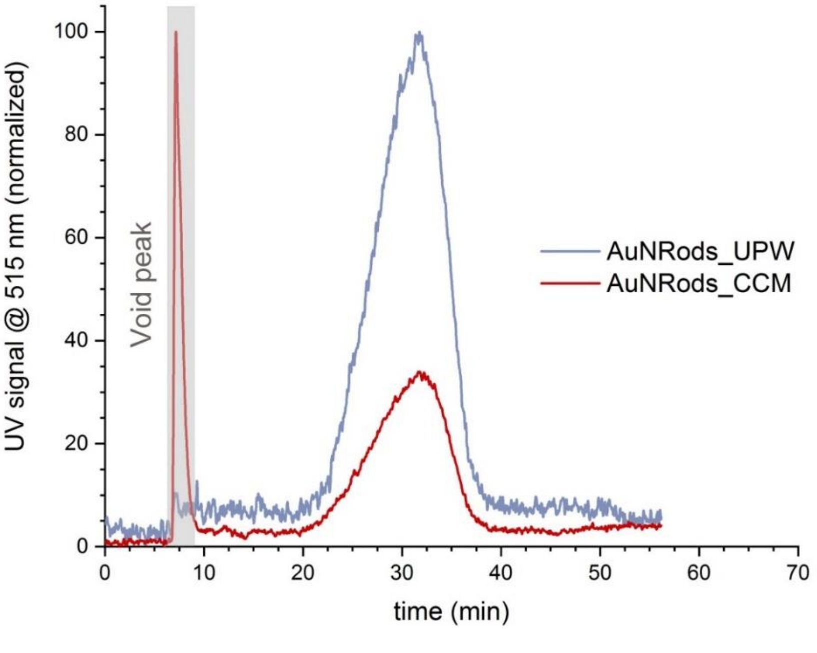

Figure S8. Fractogram with the UV/Vis signals of AuNRods comparing the sample in UPW and CCM. UPW = ultrapure water, CCM = cell culture medium.
